# Mastrevirus Rep and RepA Proteins Suppress de novo Transcriptional Gene Silencing

**DOI:** 10.3390/ijms222111462

**Published:** 2021-10-24

**Authors:** Kikyo Watanabe, Masashi Ugaki

**Affiliations:** Department of Integrated Biosciences, Graduate School of Frontier Sciences, The University of Tokyo, 5-1-5, Kashiwanoha, Kashiwa, Chiba 277-8562, Japan; ugaki@edu.k.u-tokyo.ac.jp

**Keywords:** *Geminivirus*, TYDV, transcriptional gene silencing, silencing suppressor, Rep, RepA

## Abstract

Transcriptional gene silencing (TGS) in plants is a defense mechanism against DNA virus infection. The genomes of viruses in the *Geminiviridae* family encode several TGS suppressors. In this study, we induced de novo TGS against the transgenic *GFP* gene encoding green fluorescent protein by expressing a hairpin-shaped self-complementary RNA corresponding to the enhancer region of the 35S promoter (hpE35S). In addition, we examined the TGS suppression activity of proteins encoded in the genome of *Tobacco yellow dwarf virus* (TYDV, genus *Mastrevirus*). The results show that the replication-associated protein (Rep) and RepA encoded by TYDV have TGS suppressor activity and lead to decreased accumulation of 24-nt siRNAs. These results suggest that Rep and RepA can block the steps before the loading of siRNAs into Argonaute (AGO) proteins. This is the first report of TGS suppressors in the genus *Mastrevirus*.

## 1. Introduction

Homology-dependent gene silencing (HDGS) in plants is involved in various fundamental biological processes, including defense against parasitic sequences such as transposons and viruses [1,2]. There are two major mechanisms of HDGS: transcriptional inhibition (transcriptional gene silencing, TGS), and post-transcriptional inhibition, which includes mRNA degradation and/or translational inhibition (post-transcriptional gene silencing, PTGS). The TGS is mediated by RNA-directed DNA methylation (RdDM). In plants, the RdDM pathway comprises two specific DNA-dependent RNA polymerases: Pol IV and Pol V. Pol IV transcribes 30–40 nucleotide (nt) single-stranded RNA (ssRNA), which serves as a template for RNA-dependent RNA polymerase 2 (RDR2) to produce double-stranded RNAs (dsRNAs) that are cleaved by Dicer-like3 (DCL3) into 24 nt small interfering RNAs (siRNAs) [3]. One of the two small RNA strands is incorporated into an Argonaute (AGO) protein where it base pairs with nascent transcripts of PolV and recruits DOMAINS REARRANGED METHYLTRANSFERASE 2 (DRM2) for de novo cytosine methylation on the template DNA. In this way, TGS plays a crucial role in defense against DNA viruses, including geminiviruses [4,5].

As a counter-defense measure, geminiviruses encode TGS suppressors. For example, the AC2 proteins encoded by *Cabbage leaf curl virus* (CaLCuV) and *Tomato golden mosaic virus* (TGMV), the C2 proteins encoded by *Beet curly top virus* (BCTV) and *Beet severe curly top virus* (BSCTV), and the βC1 protein encoded by *Tomato yellow leaf curl China betasatellite* (TYLCCNB) inhibit DNA methylation and suppress TGS through inhibition of the methyl cycle [6,7,8]. Similarly, the replication-associated protein (Rep) encoded by *Tomato yellow leaf curl virus* (TYLCV) can suppress TGS by downregulating host DNA methyltransferases [9]. Recent reports have suggested that many other proteins encoded in the genomes of geminiviruses, including *Tomato leaf curl Yunnan virus* (TLCYNV) C4, *Mungbean yellow mosaic India virus* (MYMIV) AC5, *Tomato yellow leaf curl Sardinia virus* (TYLCSV) Rep, *Tomato yellow leaf curl virus* (TYLCV) V2, and *Cotton leaf curl Kokhran virus* (CLCKV) Rep, TrAP, and βC1 have TGS suppression activity [10,11,12,13]. However, the mechanisms by which these proteins suppress TGS in the host plants are largely unknown.

Geminiviruses constitute a large family of plant-infecting viruses and cause severe losses in many major crops worldwide [14]. They have either bipartite or monopartite circular single-stranded DNA (ssDNA) genomes that replicate in the nucleus of an infected cell [15]. On the basis of their genome organization, host range, and insect vectors, the *Geminiviridae* family can be classified into 14 genera (with 520 species) [16].

*Tobacco yellow dwarf virus* (TYDV), which belongs to the genus *Mastrevirus*, is an important plant pathogen in Australia. TYDV is transmitted by the leafhopper *Orosius orientalis,* and it causes yellow dwarf disease in tobacco (*Nicotiana tabacum* L.) and common bean (*Phaseolus vulgaris* L.) [17,18,19,20]. Like other mastreviruses, TYDV has a monopartite, circular ssDNA genome. It replicates via a double-stranded DNA intermediate, which encodes four open reading frames (ORFs) in both virion- and complementary-sense orientations. The ORFs *V1* and *V2* on the virion-sense strand encode the coat protein (CP) and movement protein (MP), respectively [21,22,23]. Two replication-associated proteins (Rep and RepA) encoded by sequences on the complementary-sense strand are translated from differently spliced transcripts [24]. The spliced transcript that fuses the *C1* and *C2* ORFs produces Rep, whereas the unspliced transcript produces RepA from the *C1* ORF, the first ORF of the transcript. Rep is the only mastrevirus protein that is essential for viral DNA replication. Rep binds to the replication origin in a sequence-specific manner and catalyzes DNA cleavage and ligation to initiate rolling circle replication. RepA interacts with the plant retinoblastoma-related protein to make the cellular environment favorable for virus replication [25,26]. Additionally, RepA activates viral-sense transcription, possibly by direct interaction with the viral DNA [22,27]. 

Although TGS suppressor proteins have been identified in curtoviruses and begomoviruses, no such protein activity has been described for mastreviruses, including TYDV. Thus, we examined all genes encoded by the TYDV genome for their TGS suppressor activity and identified two suppressors. This suggests that the TGS mechanism is a major defensive barrier that geminiviruses must overcome.

## 2. Results

**Figure 1:**
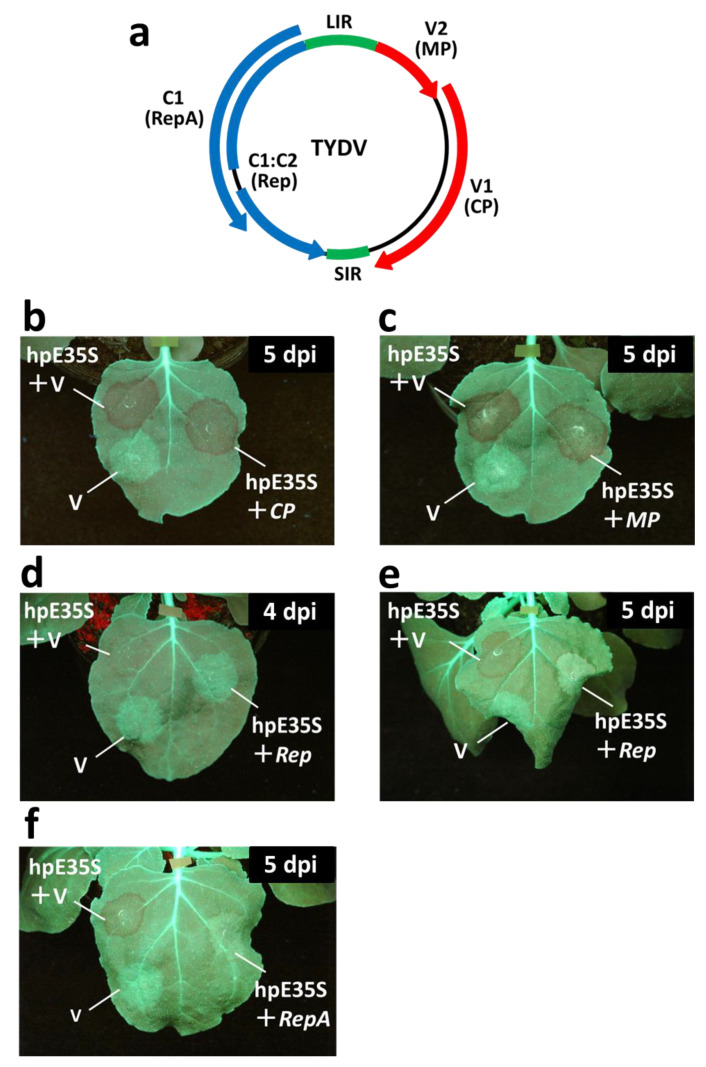
(**a**) Genome organization of *Tobacco yellow dwarf virus*. The large intergenic region (LIR) contains the origin of virion-strand replication and the bidirectional promoter regulating the expression of both virion-sense and complementary-sense genes. The small intergenic region (SIR) contains the origin of complementary-strand replication and the termination signals of the transcripts. (**b**–**e**) RNA-silencing suppression activity of genes from TYDV genome expressed in *Nicotiana benthamiana* line 16C plants. Leaves of line 16C were infiltrated with empty vector (V) or co-infiltrated with a mixture of two *Agrobacterium* transformants, one harboring hpE35S, and the other harboring either *CP* (**b**), *MP* (**c**), *Rep* (**d**,**e**), or *RepA* (**f**). Representative leaves were photographed under ultraviolet illumination at 5 days post-infiltration.

We used a transient expression system in transgenic *Nicotiana benthamiana* line 16C, which expresses GFP under the control of the *Cauliflower mosaic virus* (CaMV) 35S promoter (P35S), to analyze the TGS suppressor activity of viral genes. The regulatory region of the *GFP* gene was targeted using the hairpin-shaped self-complementary RNA corresponding to the 35S enhancer region (hpE35S) to induce RdDM and transcriptional gene silencing, leading to the loss of GFP fluorescence. The hpE35S contains an inverted repeat structure of the 35S promoter and the upstream element of the promoter (−46 to −638). The hpE35S was expressed under the control of the long NCR promoter (PLNCR), which is an extended version of the NCR promoter [28] from *Soybean chlorotic mottle virus* (SbCMV). To examine the potential TGS suppressor activity of TYDV (Genbank XXX), we tested all four genes encoded by the genome of this virus (Figure 1a) for their ability to prevent silencing of the *GFP* reporter gene in plant tissues. Technically, we followed protocols used for assays of viral PTGS suppressors [29,30]. Using this approach, a mixture of two transformants of *Agrobacterium* was infiltrated into leaves of GFP-expressing *N. benthamiana* line 16C. One transformant carried hpE35S, which initiates TGS and silences fluorescence; the other carried a candidate TGS suppressor gene or an empty vector. When the two transformants were mixed and infiltrated together, the candidate gene, if it had silencing suppression activity, was able to suppress TGS and recover visible GFP fluorescence in the infiltrated leaf area.

The empty vector (pCAMBIAΔP35S)-inoculated tissues maintained green fluorescence. At the area where hpE35S was co-infiltrated with an empty vector (“hpE35S+V” in Figure 1b–e), GFP fluorescence was greatly decreased at 4–5 days post-infiltration (dpi). This silencing of endogenous *GFP* was not affected by co-expression of the *CP* and *MP* (Figure 1b,c). On the other hand, co-expression of either *Rep* or *RepA* suppressed silencing, as indicated by the visible GFP fluorescence (Figure 1d,e).

**Figure 2:**
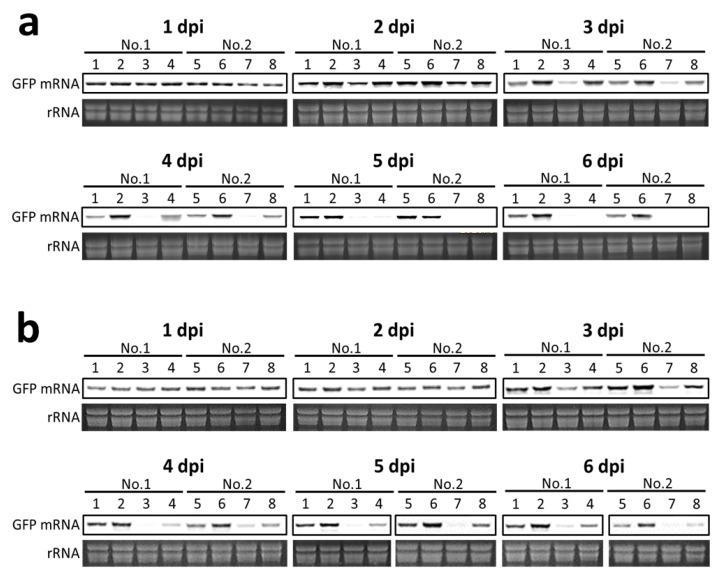
Northern blot assay of *GFP* mRNA accumulation. High molecular weight (HMW) RNA was isolated from infiltrated areas from two leaves at 1~6 days post-infiltration and subjected to northern blot hybridization analysis. rRNAs are shown as loading controls. (**a**) Analysis of *GFP* mRNA in non-infiltrated tissues (lanes 1 and 5) and tissues infiltrated with empty vector (lanes 2 and 6), or co-infiltrated with V + hpE35S (lanes 3 and 7) or hpE35S + *Rep* (lanes 4 and 8). (**b**) Analysis of *GFP* mRNA in non-infiltrated tissues (lanes 1 and 5) and tissues infiltrated with empty vector (lane 2 and 6), or co-infiltrated with V + hpE35S (lanes 3 and 7) or hpE35S + *RepA* (lanes 4 and 8).

These results were confirmed by northern blot analysis of the *GFP* mRNA (Figure 2) in the *Agrobacterium*-infiltrated areas. As shown in Figure 2a,b, at 1–2 dpi, almost similar amounts of *GFP* mRNA had accumulated in tissues infiltrated with hpE35S alone (lanes 3 and 7) or in combination of *Rep* (lanes 4 and 8 in Figure 2a) or *RepA* (lanes 4 and 8 in Figure 2b). At 4–6 dpi, *GFP* mRNA was almost undetectable in the tissues infiltrated with hpE35S alone but was still present at high levels in the tissues infiltrated with hpE35S + *Rep* at 4 dpi (lanes 4 and 8 in Figure 2a) or hpE35S + *RepA* at 4–6 dpi (lanes 4 and 8 in Figure 2b). Although the tissues infiltrated with hpE35S + *Rep* exhibited visible green fluorescence at 5 dpi, *GFP* mRNA in them was almost undetectable (Figure 1e and lanes 4 and 8 in Figure 2a). Because overexpression of mastrevirus Rep induces cell death in *N. benthamiana* at 6–10 dpi [31], autofluorescence of the damaged tissue might be observed at this time point. 

**Figure 3:**
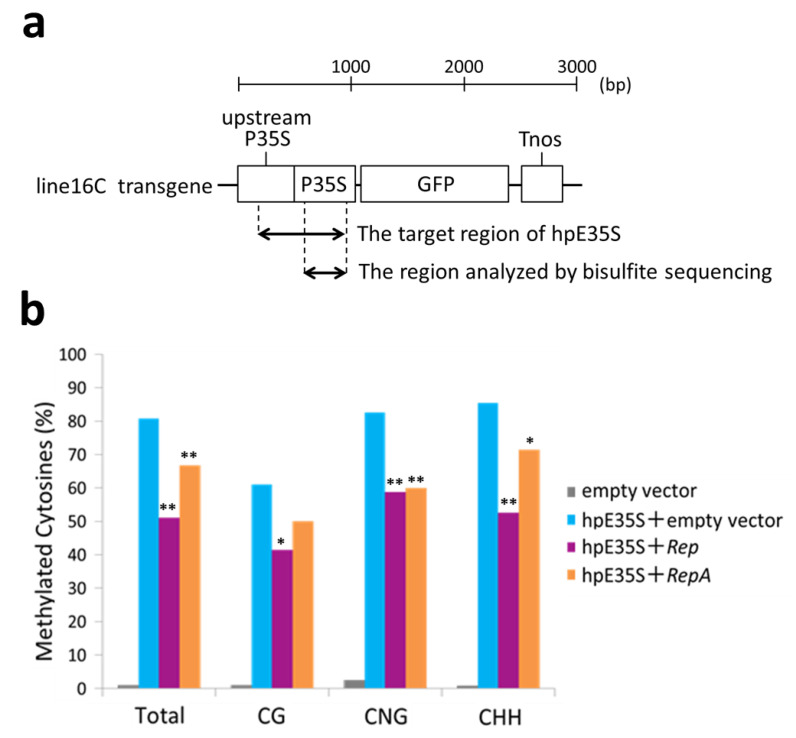
Cytosine methylation profiles in the enhancer region of 35S promoter from line 16C plants inoculated with empty vector, hpE35S + empty vector, hpE35S + *Rep*, or hpE35S + *RepA* at 5 dpi. (**a**) Schematic diagram of *Nicotiana benthamiana* line 16C transgene. The enhancer region corresponding to hpE35S and the region analyzed by bisulfite sequencing are indicated. P35S; *Cauliflower mosaic virus* (CaMV) 35S promoter, Tnos; nos terminator. (**b**) Percentage of methylated cytosine in different sequence contexts. Student′s *t*-test was performed using the methylation values from individual clones. Single and double asterisks indicate significant differences in methylation sites at *p* < 0.05 and *p* < 0.01, respectively, between hpE35S + empty vector and hpE35S + *Rep* or hpE35S + *Rep*A.

To verify the de novo DNA methylation status in the target region, we carried out bisulfite sequencing analysis using genomic DNA extracted from the infiltrated areas of 5 dpi samples. After treatment with sodium bisulfite, unmethylated cytosines were converted into uracil. Then, a part of the hpE35S target sequence was amplified by PCR and cloned, and 20 individual clones were sequenced (Figure 3a). The PCR primers were designed to hybridize cytosine-free sequences to minimize amplification bias. The analyzed sequence contained 10 CG, 4 CNG, and 41 CHH sites. In the hpE35S-infiltrated tissues, 61% of CG, 82% of CNG, and 85% of CHH sites were methylated (Figure 3b). In the *Rep* co-infiltrated tissues, 51% of CG, 42% of CNG, and 59% of CHH sites were methylated, and in the *RepA* co-infiltrated tissues, 67% of CG, 50% of CNG, and 60% of CHH sites were methylated (Figure 3b). These results show that co-expression of hpE35S with *Rep* or *RepA* reduced cytosine methylation in all sequence contexts. 

**Figure 4:**
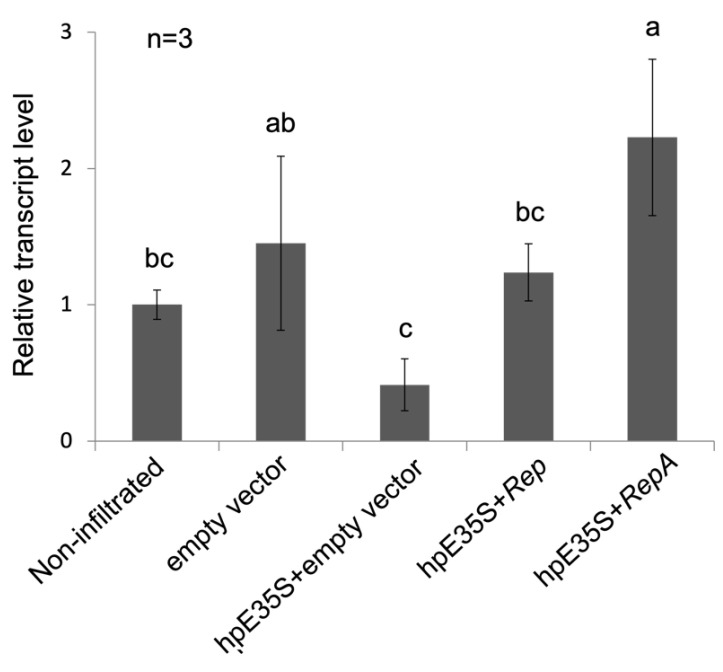
Expression level of *GFP* nascent RNA. Samples were prepared by pooling tissues from ten leaves. qPCR data were analyzed using the ΔΔCq method and internally normalized to *RubisCo* transcript level. The letters indicate statistically significant difference as determined by one-way ANOVA among multiple groups, followed by Tukey′s post hoc test.

To clarify whether or not the observed increase or decrease in *GFP* mRNA accumulation was due to regulation at the transcriptional level, we tested transcriptional activity by analyzing nascent RNAs. We isolated nuclei from infiltrated tissues and performed nuclear run-on transcription in the presence of bromouridine (BrdU), followed by isolation of BrdU-labeled nascent transcripts for detection by RT-qPCR. The expression of hpE35S caused a significant decrease in the *GFP* transcriptional rate (see “hpE35S + empty vector” in Figure 4), while co-expression of hpE35S with *Rep* or *RepA* suppressed transcription (Figure 4). These results suggested that the Rep and RepA proteins have TGS suppressor activity. 

**Figure 5:**
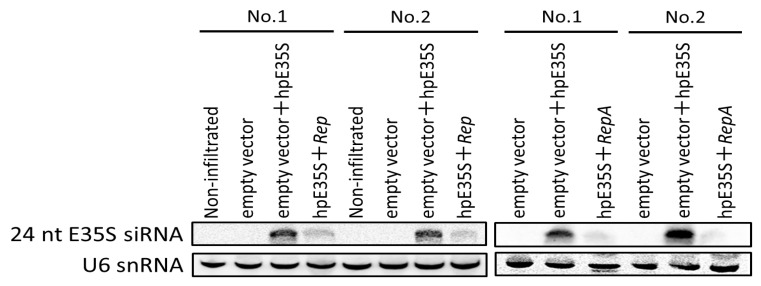
Analysis of E35S-specific siRNAs in non-infiltrated tissues and tissues infiltrated with empty vector or co-infiltrated with a mixture of two *Agrobacterium* transformants, one harboring hpE35S, and the other harboring either V, *Rep*, or *RepA* (top panel). Low molecular weight (LMW) RNA was isolated from infiltrated zones at 5 days post-infiltration and subjected to northern blot hybridization analysis. Two samples were prepared by pooling tissues from seven leaves. U6 snRNA served as the loading control (bottom panel).

To gain a better insight into how *Rep* and *RepA* suppress TGS, we examined whether they prevent the accumulation of the siRNAs that induce TGS. As shown in Figure 5, 24 nt siRNAs were readily detected in the hpE35S-infiltrated tissues at 5 dpi, when RNA silencing had been established (Figure 2), while their abundance was decreased by co-infiltration with *Rep* or *RepA*. Equal loading of RNA samples was assessed by sequential stripping and re-probing of the blot using a probe specific to U6 snRNA.

## 3. Discussion

Most of the previously reported geminivirus-encoded TGS suppressors act through interfering with the host DNA methylation machinery [6,7,8,9]. For example, the Rep protein of TYLCSV (genus *Begomovirus*) interferes with DNA methyltransferases. In this study, we showed that *Rep* and *RepA* of TYDV (genus *Mastrevirus*) can reduce the accumulation of 24 nt siRNAs, inhibit de novo methylation of cytosine residues in all sequence contexts in the enhancer region of the 35S promoter, and suppress the transcription of *GFP* mRNA. These results suggested that Rep and RepA proteins function as TGS suppressors and inhibit steps upstream of the loading of siRNAs into AGO. Thus, the Rep proteins encoded by begomoviruses and mastreviruses have distinct functional mechanisms to suppress TGS. Previously, Rep and RepA from another mastrevirus, *Wheat dwarf virus* (WDV), were shown to have PTGS suppression activity. They were found to function similarly to P19 encoded by tombusviruses and HC-Pro encoded by potyviruses [32,33], which sequester siRNAs and diminish their accumulation [34]. However, TGS suppressors that function via such mechanisms have not yet been identified. Therefore, our results offer new insights into the functions of TGS suppressors encoded by geminiviruses. 

This report identifies TGS suppressors of a geminivirus in the genus *Mastrevirus*. Interestingly, although the TYDV genome encodes only four genes, our data revealed that two of them encode TGS suppressors. Because Rep and RepA proteins are translated from partially overlapping ORFs, they share approximately 200 identical amino acid residues at the N-terminus [35]. Therefore, this region is likely to contain the domain involved in TGS suppressor activity. The common region includes a DNA-binding domain, cleavage and ligation domains for rolling circle replication, an oligomerization domain, and a retinoblastoma-related protein-binding domain. Hence, the functions of one or more of these domains might be related to TGS suppressor activity.

Although the TGS mechanism was initially discovered as a response to virus infection, it has since been shown to play important roles in the regulation of plant growth and the response to other biotic stresses [36]. Thus, the discovery of a new viral TGS suppressor may open a new avenue to understand these mechanisms. In addition, these findings can be applied to genetic engineering in plants, where it is desirable to manipulate gene silencing.

## 4. Materials and Methods

### 4.1. Plant Material and Growth Conditions

The transgenic *N. benthamiana* line 16C expressing GFP has been described previously [37]. The plants were cultivated in growth chambers at 25 °C under a 16 h light/8 h dark photoperiod with light supplied by cool fluorescent lamps at ~100 µmol m^−2^ s^−1^.

### 4.2. Construction of hpE35S and Gene Expression Vectors

For the construction of hpE35S, the E35S fragment was first amplified from line 16C genomic DNA using the primer pairs E35S-F and E35S-R. The fragment was then digested with *Sac*I and *Xba*I, and cloned into pUC19 vector to construct pUC-E35S. pUC-E35S was digested with *BamH*I and *Sac*I, and the E35S fragment was cloned into pE7133 plasmid [8] to construct pE7133-anti E35S. Then, the PLNCR was amplified from the SbCMV genome with the primers PLNCR-F and PLNCR-R, digested with *Hind*III and *SnaB*I, and cloned into pE7133-anti E35S to construct pE7133-PLNCR-anti E35S. Then, pUC-E35S was digested with *Sma*I and *Xba*I, and the E35S fragment was cloned into pE7133-PLNCR-anti E35S digested with *SnaB*I and *Xba*I to construct pE7133-PLNCR-hpE35S. The RbcS terminator of the pSMAK760 plasmid was cloned into *Sac*I-*EcoR*I-digested pE7133-PLNCR-hpE35S. Finally, the *EcoR*I-*Hind*III region, including the expression cassette, was cloned into the binary vector pCAMBIA2300ΔP35S so that the CaMV 35S promoter region was removed to avoid gene silencing. 

To construct the TYDV gene expression vectors, each gene was amplified from an infectious clone of TYDV by PCR and cloned into the *BamH*I and *Sac*I sites of the pE7133 plasmid to construct pE7133-TYDV ORF. Then, the RbcS terminator of the pSMAK760 plasmid was cloned into *Sac*I-*EcoR*I digested pE7133-TYDV ORF. Finally, the *EcoR*I-*Hind*III region, including the expression cassette, was cloned into the binary vector pCAMBIA2300ΔP35S. For the *C1 (RepA)*, *V1 (CP)*, and *V2 (MP)* genes, PCRs were performed with the primer pair TYDV-RepA-F/TYDV-RepA-R, TYDV-CP-F/TYDV-CP-R, and TYDV-MP-F/TYDV-MP-R, respectively. For the *Rep* gene, two independent PCRs were performed with the primer pair TYDV-Rep-UF/TYDV-Rep-UR and TYDV-Rep-DF/TYDV-Rep-DR. The PCR products were fused and amplified using overlap extension-PCR as described previously [38,39]. All PCRs were performed using a high-fidelity KOD-plus-DNA Polymerase (TOYOBO, Osaka, Japan), and their products were verified by DNA sequencing. The primer sequences are listed in Supplementary Table S1.

### 4.3. Agroinfiltration of Leaves and GFP Imaging

The binary plasmids described above were introduced into the *Agrobacterium tumefaciens* strain EHA105 [40] by the freeze–thaw transformation method, and the resulting bacteria were cultured on solid LB medium containing 50 µg/mL kanamycin for 2 days at 28 °C. A single colony of *Agrobacterium* was inoculated and cultured in LB medium containing 50 µg/mL kanamycin for 20 h at 28 °C. Bacterial cultures were diluted 1:100 in fresh LB medium containing 50 µg/mL kanamycin and grown to an optical density A_600_ = 0.5. The cultures were resuspended in equal volume of infiltration medium containing 10 mM MgCl_2_, 10 mM MES (pH 5.6), and 150 µM acetosyringone, and incubated for 12 h at 28 °C. For patch infiltration assays, the corresponding bacterial cultures were mixed with equal volume ratios and infiltrated into young, fully expanded leaves of 4-week-old plants using a 1 mL needleless syringe. Then, GFP fluorescence in plant leaves was observed using a handheld long-wave ultraviolet lamp (366 nm; BLAK-RAY Model UVL-56, San Gabriel, CA, USA) and photographed with a digital camera (Canon DS6031, Canon, Tokyo, Japan) with a low-cut filter (Kenko Y2: cut under 480 nm; Kenko Tokina Co., Ltd., Tokyo, Japan). To analyze transient expression, samples were collected at 5 dpi. All inoculations were repeated a minimum of three times in independent experiments.

### 4.4. Northern Blot Analysis

For northern blotting analyses, total RNA was isolated from agroinfiltrated leaves using TRI Reagent (Cosmo Bio Co., Ltd., Tokyo, Japan). For *GFP* mRNA blotting, high molecular weight (HMW) RNA was enriched from total RNA by eliminating low molecular weight (LMW) RNA using 10M LiCl. Then, 5 µg HMW RNA diluted with loading dye was heated at 65 °C for 10 min, separated by electrophoresis in 1.8% formaldehyde gels, and transferred onto Hybond N+ membranes using a capillary transfer system. For siRNA blotting, LMW RNA was enriched from total RNA by eliminating HMW RNA using 13% polyethylene glycol (8000 MW) in 1.6 M NaCl. Then, 10 µg LMW RNA diluted with loading dye was heated at 95 °C for 2 min and separated by electrophoresis in 15% polyacrylamide gels (19:1 ratio of acrylamide to bis-acrylamide, 8 M urea), and transferred onto Hybond N+ membranes using a semi-dry electroblotting apparatus (Bio-Rad, Hercules, CA, USA) in 1× TBE buffer at 100 mA for 1 h. The HMW and LMW blots were hybridized with digoxigenin (DIG)-labeled DNA probes specific to the *GFP* gene and E35S, respectively (DIG PCR labeling kit, Roche, Basel, Switzerland). After hybridization, membranes were washed with wash buffer 1 (2× SSC, 0.1% SDS) at room temperature and then with wash buffer 2 (0.2× SSC, 0.1% SDS) at 50 °C. Hybridization signals were detected after treatment with CDP-Star (Roche, Mannheim, Germany) using a Gel Documentation system LAS 4000 (Fuji Photo Film Co., Tokyo, Japan). To confirm equal loading, ethidium bromide staining of RNA before transfer and detection of U6 snRNA were conducted.

### 4.5. Bisulfite Sequencing

Genomic DNA was extracted from the leaf samples using the CTAB method [41]. Then, 300 ng of total DNA was subjected to bisulfite modification with the EZ DNA Methylation-Gold kit (Zymo Research, Irvine, CA, USA). Bisulfite-modified DNA was purified and dissolved in a 10 µL elution buffer according to the manufacturer′s instructions. The PCRs were then performed using TaKaRa EpiTaq^TM^ HS for bisulfite-treated DNA (TaKaRa Biomedical, Otsu, Japan). We selected the PCR primers E35S Bisul 11 and E35S Bisul 12 (Supplementary Table S1) to amplify a 448-bp fragment, which contained the target region of hpE35S. The amplified products were cloned into the pUC18 vector. Then, cloned DNA fragments were sequenced with the ABI 3500 Genetic Analyzer (Applied Biosystems, Foster City, CA, USA) and analyzed using Kismeth software (http://katahdin.mssm.edu/kismeth/revpage.pl, accessed on 1 September 2021) [42] to quantify cytosine methylation levels (20 independent clones for each sample). To ensure that the bisulfite modification was complete, methylated and unmethylated control plasmids were added to each sample before bisulfite treatment. 

### 4.6. Nuclear Run-on Assay

Plant tissue (approx. 500 mg to 1 g) was ground into a fine powder in liquid nitrogen and suspended in a 10 mL lysis buffer (20 mM Tris-HCl pH 7.5, 20 mM KCl, 2 mM EDTA pH 8.0, 2.5 mM MgCl_2_, 25% glycerol, 250 mM sucrose, Roche protease inhibitor cocktail). After centrifugation at 1000× *g* for 15 min at 4 °C, the pellet was resuspended in a 10 mL extraction buffer (20 mM Tris-HCl pH 7.5, 2.5 mM MgCl_2_, 25% glycerol, 0.2% Triton X-100, Roche protease inhibitor cocktail), and the mixture was centrifuged at 1000× *g* for 15 min at 4 °C to pellet the nuclei. Then, the pellet was resuspended in 200 µL nuclei storage buffer (50 mM Tris-HCl pH 8.3, 0.1 mM EDTA, 5 mM MgCl_2_, 40% glycerol). Nuclear run-on reactions were performed in the presence of 5-bromouridine 5′-triphosphate (BrUTP), and nascent transcripts labelled with BrUTP were immunoprecipitated as described previously [43]. The RNA was extracted using a TRI reagent-based protocol following the manufacturer′s recommendations. cDNA synthesis was performed using PrimeScript RT reagent kit (Perfect Real Time; Takara Bio Inc., Shiga, Japan). RT-qPCR analyses were performed with the Thermal Cycler DiceTM Real Time System (Takara, Tokyo, Japan) using THUNDERBIRD SYBR qPCR Mix (Toyobo, Osaka, Japan). The transcript level of *GFP* was normalized to that of *RubisCo* in each sample. The gene transcript data were analyzed using the 2^−ΔΔCT^ method. The primers used are listed in Supplementary Table S1. 

## Data Availability

Not applicable.

## Supplementary Materials

The following are available online at https://www.mdpi.com/article/10.3390/ijms222111462/s1.
